# The Alleged Infecundity of Females Born Co-Twins with Males

**Published:** 1844-02-24

**Authors:** 


### THE ALLEGED INFECUNDITY OF FEMALES BORN CO-TWINS WITH MALES.

Dr. Simpson, in a very interesting paper on this subject, concludes—

"1. That, in the human subject females born co-twins with males, are, when married, as likely to have children as any other females belonging to the general community.

"2. That, when they are married and become mothers, they are, in respect to the number of their children, as productive as other females.

"3. That the same law of the fecundity of the female in opposite sexed twins seems to hold good among all our uniparous domestic animals, with the *exception* of the cow alone."—*Ed. Med. and Surg. Journ.*

